# Atypical cancer pattern in patients with Parkinson's disease

**DOI:** 10.1038/sj.bjc.6602279

**Published:** 2004-12-07

**Authors:** J H Olsen, S Friis, K Frederiksen, J K McLaughlin, L Mellemkjaer, H Møller

**Affiliations:** 1 Institute of Cancer Epidemiology, Danish Cancer Society, Copenhagen, Denmark; 2 International Epidemiology Institute, Rockville, MD, USA; 3 Thames Cancer Registry, King's College London, London, UK; 4 London School of Hygiene & Tropical Medicine, London, UK; 5 Vanderbilt University Medical Centre, Vanderbilt-Ingram Cancer Center, Nashville, TN, USA

**Keywords:** Parkinson's disease, cancer incidence, malignant melanoma, nonmelanocytic skin cancer, breast cancer

## Abstract

Among 14 088 patients, with a primary diagnosis of Parkinson's disease during the period 1977–98 identified from the National Register of Patients, 1282 cancers were subsequently recorded in the Danish Cancer Registry, compared with 1464 expected, with a standardised incidence ratio (SIR) of 0.88 (95% confidence interval (CI), 0.8–0.9). Significantly reduced risks were found for smoking-related cancers, for example, cancers of the lung (SIR, 0.38), larynx (0.47) and urinary bladder (0.52), although moderate reductions in risk were also seen for several nonsmoking-related cancers. In contrast, increased risks were seen for malignant melanoma (SIR, 1.95; 95% CI, 1.4–2.6), nonmelanocytic skin cancer (1.25; 1.1–1.4) and breast cancer (1.24; 1.0–1.5). The observed cancer pattern supports the hypothesis that constituents of tobacco smoke inhibit or delay the development of Parkinson's disease, but a low smoking prevalence appears to be only part of the explanation for the decreased cancer incidence. The increased relative risks of melanoma and nonmelanoma skin cancer are not likely to be artefactual, but further investigations of potential mechanisms are warranted.

Parkinson's disease is a movement disorder of middle or late life, characterised by an abnormal loss of pigmented neurons of unknown cause and mechanism in the substantia nigra of the brain stem. As early as in 1954, Doshay noted that ‘for reasons as yet unclear, cancer is phenomenally rare in paralyses agitans’ (Doshay, 1954). Formal cohort studies of cancer incidence (Jansson and Jankovic, 1985; Møller *et al*, 1995; Minami *et al*, 2000) and cancer mortality (Ben-Shlomo and Marmot, 1995; Vanacore *et al*, 1999; Minami *et al*, 2000) in patients with Parkinson's disease subsequently confirmed the overall pattern of a reduced risk. The risk reduction has been related to the relatively consistent observation that a high proportion of individuals who later develop Parkinson's disease are lifelong nonsmokers, as compared with the general population (Hernan *et al*, 2002). However, a few specific cancer types have been reported to occur in excess of expected numbers, including cancers of the thyroid and the female breast (Jansson and Jankovic, 1985; Møller *et al*, 1995; Minami *et al*, 2000) and malignant melanoma of the skin (Jansson and Jankovic, 1985; Møller *et al*, 1995). Treatment of Parkinson's disease with levo-dopa has been suggested as a causal factor in the latter association, because levo-dopa serves as a substrate for the enzyme tyrosine hydroxylase, which is involved in the production of dopamine and melanin (Kincannon and Boutzale, 1999; Fiala *et al*, 2003).

Here, we report the results of an extension of a Danish cohort study (Møller *et al*, 1995) of cancer in patients with Parkinson's disease.

## METHODS

The study cohort was identified from the files of the Danish National Hospital Register, which was instituted on 1 January 1977 and contains information on all nonpsychiatric hospital admissions in Denmark (Andersen *et al*, 1999). Since 1 January 1994, it has been supplemented by information on outpatient visits to somatic disease departments. Each admission or outpatient visit initiates a record flagged by the personal identification number of the patient, a number which incorporates sex and date of birth and which permits accurate linkage between nationwide registers.

The study covered 14 502 patients whose admission or outpatient records for 1977–98 included a primary diagnostic code for Parkinson's disease (ICD-8 code 342 and ICD-10 code G20). For patients who had been referred more than once to hospital or outpatient clinic with a diagnosis of Parkinson's disease, only the first record was retained and provided the date of entry into the study. Cohort members were linked to the Danish National Mortality Files in order to verify vital status through 1999. A total of 32 patients (0.2%) were excluded from the cohort because their identification number indicated that they were citizens of the Faroe Islands or Greenland. A further 369 (2.9%) were excluded because they had died during their stay in hospital. We also chose to exclude 13 patients (0.1%) who were discharged with a diagnosis of Parkinson's disease when they were under the age of 20. These exclusions left a cohort of 14 088 patients for the analysis of subsequent cancer incidence, comprising 12 803 seen as in-patients during the period 1977–98 and 1285 seen only as outpatients during the period 1994–98 (Table 1). The age-specific and age-standardised rates of Parkinson's disease were calculated for men and women separately per 100 000 individuals seen as in-patients and out-patients. The age composition of the Danish population in 1987, totalling 5 127 070, was used as the standard.

Cohort members were linked to the files of the Danish Cancer Registry in order to ascertain incident cases of cancer. The Cancer Registry, which began reporting incidence data on a nationwide scale in 1943, includes cases of nonmelanocytic skin cancer (basal cell as well as squamous cell carcinoma), benign brain tumour and bladder papilloma under cancers of the skin, brain and bladder, respectively (Storm *et al*, 1997). The follow-up period for cancer began on the date of first hospital discharge or outpatient visit with a primary diagnosis of Parkinson's disease and ended on the date of death (*n*=10 648) or on 31 December 1999 (*n*=3440), whichever occurred first (Table 1). The number of cancers observed was compared with the numbers expected, which were calculated by multiplying the number of person-years for cohort members by the overall and site-specific incidence rates of cancer for Danish men and women in 5-year age groups and calendar periods of observation. Standardised incidence ratios (SIRs) for cancer in patients with Parkinson's disease, and 95% confidence intervals (CIs) were calculated, assuming a Poisson distribution of the observed cancers (Bailar and Ederer, 1964). Multiple primary cancers in the same patient during follow-up were included in observed as well as expected numbers.

To evaluate possible misclassification in the Danish Cancer Registry of lentigo maligna or level I melanomas (which are probably pre-invasive melanomas) as invasive melanomas, we reviewed the original notification forms of all patients with Parkinson's disease who were registered with a malignant melanoma (*n*=44) and of an age- and sex-matched sample of approximately four times as many individuals from the general population registered in the Cancer Registry with malignant melanoma (*n*=168).

## RESULTS

The 14 088 patients in this study had an average age at first hospital admission or outpatient visit for Parkinson's disease of 72.8 years and accrued 69 802 person-years of follow-up (average, 5.0 years; range, up to 23 years). Almost 13% of the patients were followed up for at least 10 years. Table 2 gives the age-standardised and age-specific incidence rates of a primary diagnosis of Parkinson's disease for inhabitants of Denmark during the recruitment period 1977–98. The rates for the first years of registration are somewhat inflated by inclusion of prevalent cases of Parkinson's disease, as some of the patients might have had the disease for several years before the start of the National Hospital Register in 1977. After 1994, an increasing number of cases were diagnosed in outpatient clinics. During the 1990s, a steep increase in the age-standardised incidence was seen among men, that is, from 17 per 100 000 in 1989–91 to 28 per 100 000 in 1998, with a modest increase among women over the same period (Table 2). In the late 1970s, the male : female ratio for the disease was 1.2, whereas in the late 1990s it was 1.9. Table 2 shows that the sex difference in incidence grew with increasing age.

Overall, 1282 cancers were reported, whereas 1463.9 were expected, with a SIR of 0.88 (95% CI, 0.8–0.9), equivalent to a significant cancer deficit of 12% (Table 3). Cancers at a few sites occurred more frequently than in the general population, including malignant melanoma (SIR, 1.95; 1.4–2.6), nonmelanocytic skin cancer (1.25; 1.1–1.4) and cancers of the breast (1.24; 1.0–1.5), brain (1.32; 0.9–1.9) and at unspecified or secondary sites (1.21; 1.0–1.5). Excluding malignant melanoma and nonmelanocytic skin cancer generated a 19% deficit (SIR, 0.81; 0.8–0.9). The overall decreased risk was mainly due to a markedly reduced risk for smoking-related cancers (combined SIR, 0.58; 0.5–0.6). In particular, reductions were seen for cancers of the lung (SIR, 0.38), larynx (0.47) and urinary bladder (0.52), which are the cancers most strongly associated with tobacco smoking. Nevertheless, lower rates of several other site-specific cancers presumably not related to tobacco smoking were also seen, most notably cancers of the prostate (0.74), colon (0.84) and rectum (0.89).

**Figure 1:**
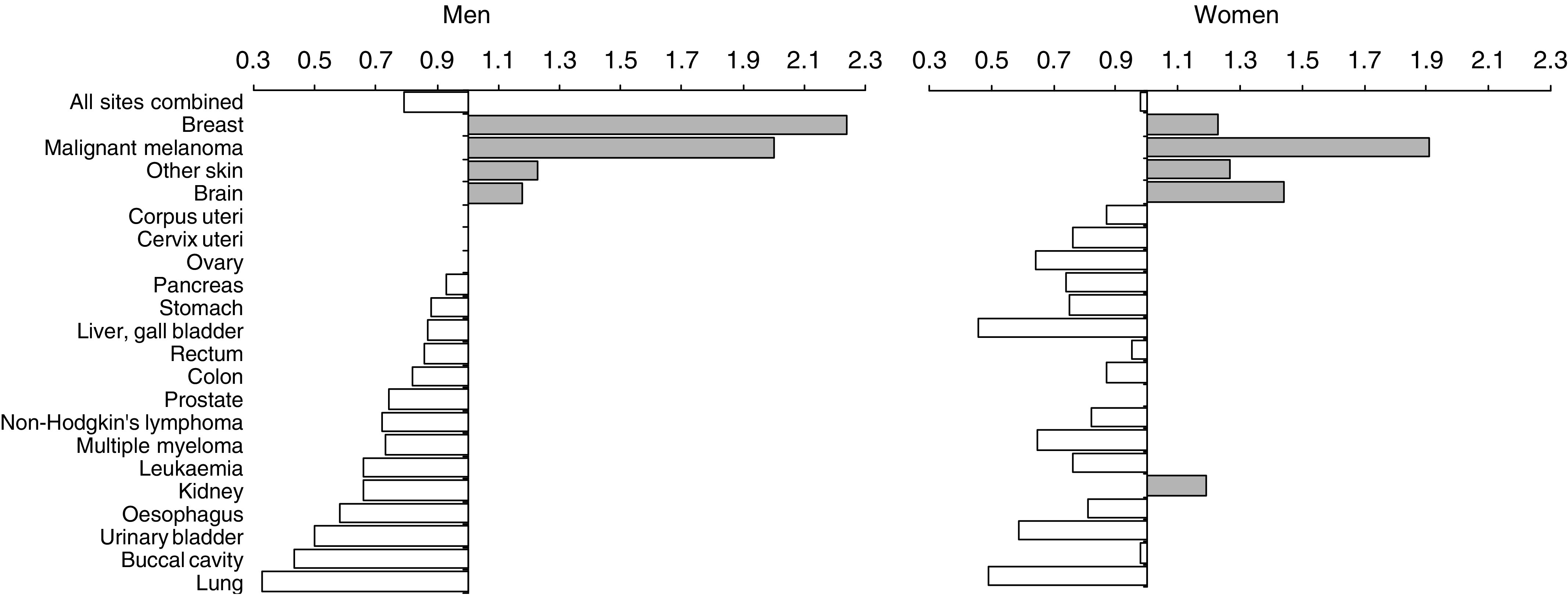
Relative risks for cancers at all sites combined and for individual sites, in men and women with Parkinson's disease in Denmark, 1977–98.

The risk for all cancers was significantly reduced for men (SIR, 0.79; 0.7–0.9), but not for women (0.98; 0.9–1.1) (Figure 1). The risks for cancer at individual sites, however, were similar for men and women, for example, SIRs of 0.33 and 0.65 for lung cancer, 0.50 and 0.59 for bladder cancer, 2.00 and 1.91 for malignant melanoma and 1.23 and 1.27 for nonmelanocytic skin cancer, respectively. Kidney cancer was the only exception, with SIRs for men and women of 0.66 (0.4–1.1) and 1.19 (0.7–1.9), respectively, on the basis of 14 and 19 observed cancers. The difference in the sex-specific overall risk was mainly due to the fact that smoking represents a smaller burden of cancer in women than in men, that the excess risk for breast cancer has a comparatively larger impact in women, and that a marked deficit was found among men for prostate cancer.

In a further analysis by time since entry (Table 4), there was a tendency for an increasing risk reduction with increasing length of follow-up for cancer overall and for most site-specific cancers, both smoking and nonsmoking related. The trend for breast cancer in women, however, showed clearly increasing estimates with increasing length of follow-up. The risk of malignant melanoma was particularly high during the first 5 years of follow-up. The excess risk for brain cancer seen initially might be at least partly due to diagnostic misclassification, if early symptoms of brain cancer were wrongly diagnosed as a sign of Parkinson's disease, or detection bias, if brain cancers were diagnosed during the clinical work-up for Parkinson's disease.

### Malignant melanoma of the skin

We reviewed the Cancer Registry notification forms for 43 of 44 cohort members registered with a malignant melanoma (one patient was notified on the basis of a death certificate only) and the notification forms of a random sample of 168 individuals likewise registered with a malignant melanoma. ‘Lentigo maligna’ or ‘level I melanoma’ (regarded as pre-invasive) appeared on the notification forms of three (7%) of the 43 cohort members and 16 (10%) of the 168 individuals in the random sample. The severity of the lesion was not given for 27% of the cohort members and for 23% of the sample, while invasiveness was clearly stated on the notification forms of 66% of the cohort members and 67% of the sample.

## DISCUSSION

In this population-based study of more than 14 000 patients with a primary diagnosis of Parkinson's disease, we observed a statistically significant overall 42% decrease in the risk for cancer at sites related to tobacco smoking. The decrease was most pronounced for men and for the smoking-related sites of lung, larynx and urinary bladder, suggesting that the risk reduction was probably due to a low prevalence of smoking. Large prospective studies have uniformly shown that the incidence of Parkinson's disease is about 60% lower among current smokers and 40% lower among past smokers than among those who have never smoked, and that the magnitude of the risk reduction diminishes with time since quitting smoking (Hernan *et al*, 2002, 2001). Similarly reduced risk estimates for Parkinson's disease linked with cigarette smoking were observed in large, well-conducted case–control studies (Gorell *et al*, 1999; Checkoway *et al*, 2002). These findings led to the hypothesis that constituents of tobacco smoke can reduce or abolish the brain damage that triggers Parkinson's disease. This hypothesis of a true protective effect of tobacco smoke is further supported by the recent observation that the risk for the disease in both dizygotic and monozygotic twins is inversely associated with the life-long dose of cigarette smoke, measured in pack-years (Tanner *et al*, 2002).

The descriptive epidemiology of Parkinson's disease in Denmark appears at least partly to accord with a protective effect of tobacco smoke: the smoking habits of Danish women have been stable or perhaps increased slightly over the past three decades, and their incidence of Parkinson's disease is largely unchanged, while the smoking habits of Danish men decreased over the same period, which is consistent with their increased incidence of the disease (Skuladottir *et al*, 2000; Juel, 2002). The observation that the overall rate of Parkinson's disease is higher in men than in women, however, is not explained by changes in smoking habits in the general population.

With the clear exception of cancers of the skin and breast, our study also showed that the incidence of nonsmoking-related cancers in the cohort was 19% lower than that of the general population, although significant reductions were seen only for cancers of the prostate and colon. We have no ready explanation for the decreased risks for most nonsmoking-related cancers. A negative surveillance bias might have arisen from the relatively poor communication ability of the patients most seriously affected by their movement disorder, as certain malignancies might have been missed in such patients, leading to a reduced number of notifications to the Cancer Registry. This is, however, unlikely to explain the overall low incidence, as the risk reduction was seen from the date of the first notification of Parkinson's disease. Moreover, the significant excesses of cancers of the breast and skin speak against a general under-diagnosis of cancer in this patient group. If the observed decreases in the risks for most nonsmoking-related cancers are not due to bias, they suggest other aetiologic hypotheses. If it is assumed that apoptosis in the substantia nigra is a manifestation of the apoptotic potential of the patient in general, the development of Parkinson's disease might be considered a marker for an individual's ability to induce cell death by apoptosis, that is, to provide protection against the effects of mutagenic exposures, including to tobacco and other chemical carcinogens.

The increased relative risk for skin cancer was so marked in our study (about 25% for nonmelanocytic skin cancer, based on 292 observed cases, and two-fold for malignant melanoma, based on 44 cases) that it is unlikely to be completely explained by better medical surveillance of these patients. Moreover, as a serious cancer, it is likely at some time to be diagnosed and notified to the Cancer Registry. Our review of the notifications of patients with Parkinson's disease and malignant melanoma did not confirm the suspicion that pre-invasive cases of malignant melanoma are more often reported to the Cancer Registry for patients with Parkinson's disease than for Danish residents in general. Solar radiation is the main cause of both malignant melanoma and nonmelanocytic skin cancer (International Agency for Research on Cancer, 1992). In a study of avoidable cancers in the Nordic countries, it was estimated that 85–90% of all malignant melanomas diagnosed are caused by solar radiation (Olsen *et al*, 1997), and similar estimates for nonmelanocytic skin cancer have been published (Harvard Center for Cancer Prevention, 1996). We have no reason to believe that individuals who develop Parkinson's disease also belong to the subgroup of the population who has above-average participation in outdoor activities, with ambient exposure to sunlight and episodes of sunburn. The specific hypothesis of a causal association between malignant melanoma (and possibly other skin cancers) and treatment with levodopa is currently under debate (Fiala *et al*, 2003) and could not be evaluated in the present study as we had no systematic information on the treatment received by the patients over the years.

We have no explanation for our finding of a marginally significant, 23% increase breast cancer, based on a total of 139 observed cases. An excess of incident cases of breast cancer was also seen in a smaller Japanese study (Minami *et al*, 2000). In contrast, a decreased mortality from breast cancer was seen in an Italian study, based on 29 cases. We had no information on the reproductive factors that are important potential confounders in studies of female breast cancer, although these factors are unlikely to be associated with Parkinson's disease.

Patients with less severe disease were potentially missing from our cohort, implying that the atypical cancer pattern of patients with Parkinson's disease is not fully representative of that of all patients with the disease, although the inclusion of outpatient diagnosed patients may have ameliorated this potential shortcoming. Our results point to the need for hypotheses to explain why patients with Parkinson's disease are protected from most cancers but at the same time are at increased risk for melanoma and non-melanoma skin cancer, and perhaps breast cancer.

## Figures and Tables

**Table 1:** The distribution of patients with a diagnosis of Parkinson's disease during 1977–1998 by sex, year of birth, type of patient and vital status on 31 December 1999 (end of follow-up)

**Characteristic**	**Men (%)**	**Women (%)**	**Both sexes (%)**
All patients	7190 (100)	6898 (100)	14 088 (100)
			
*Year of birth*			
<1900	412 (6)	466 (7)	878 (6)
1900–1919	4674 (65)	4723 (68)	9397 (67)
1920–1939	1901 (26)	1576 (23)	3477 (25)
1940–1959	196 (3)	128 (2)	324 (2)
⩾1960	7 (<1)	5 (<1)	12 (<1)
			
*Patient type*			
Inpatient	6474 (90)	6329 (92)	12 803 (91)
Outpatient^a^	716 (10)	569 (8)	1285 (9)
			
*Vital status*			
Alive	1602 (22)	1838 (27)	3440 (24)
Deceased	5588 (78)	5060 (73)	10 648 (76)

a On the basis of outpatient visits only.

**Table 2:** Age-standardised and age-specific rates of Parkinson's disease (PD) in Denmark per 100 000 men (*n*=7190) and women (*n*=6898)

	**Men**			**Women**			
**Characteristic**	**Inpatient**	**Outpatient**	**All**	**Inpatient**	**Outpatient**	**All**	**Both sexes (all)**
*Period* a							
1977–1979	23.1		23.1	18.5		18.5	20.4
1980–1982	21.3		21.3	16.7		16.7	18.5
1983–1985	19.3		19.3	14.1		14.1	16.2
1986–1988	17.5		17.5	11.5		11.5	13.8
1989–1991	17.0		17.0	12.4		12.4	14.2
1992–1994	16.7	4.8	18.3	10.4	2.6	11.3	14.0
1995–1997	16.8	8.9	25.7	10.2	5.3	15.3	19.5
1998	15.8	11.7	27.6	8.5	6.1	14.6	19.9
							
*Age at first discharge (years)*							
20–34	0.1	0.0	0.1	0.1	0.0	0.1	0.1
35–49	1.0	0.2	1.2	0.9	0.1	1.0	1.1
50–64	10.7	1.5	12.2	8.5	0.9	9.4	10.8
65–79	68.4	6.8	75.2	53.5	4.1	57.6	65.4
⩾80	110.2	13.1	123.3	57.5	6.9	64.4	84.3

a Period of first inpatient registration (1977–1998), or if no inpatient registration first outpatient registration (1994–1998).

**Table 3:** Standardised incidence ratios (SIR) for total and site-specific cancers among 14 088 patients with a primary diagnosis of Parkinson's disease, 1977–1998

**Site of cancer (ICD-7)**	**No. observed**	**No. expected**	**SIR**	**95% CI**
All sites combined (140–205)	1282	1463.9	0.88	0.8–0.9
Melanoma of skin (190)	44	22.5	1.95	1.4–2.6
Other skin (191)	292	234.4	1.25	1.1–1.4
All sites except skin	990	1229.5	0.81	0.8–0.9
Breast	142	114.6	1.24	1.0–1.5
All sites except skin and breast	804	1092.4	0.74	0.7–0.8
				
*All smoking-related sites*	285	491.3	0.58	0.5–0.6
Lung (162.0–1)	66	174.8	0.38	0.3–0.5
Larynx (161)	5	10.7	0.47	0.2–1.1
Urinary bladder (181)	50	95.3	0.52	0.4–0.7
Buccal cav., pharynx (140–148)	15	24.6	0.61	0.3–1.0
Esophagus (150)	10	15.2	0.66	0.3–1.2
Myeloid leukaemia (204, partly)	10	14.4	0.69	0.3–1.3
Liver	10	14.4	0.70	0.3–1.3
Cervix uteri (171)	10	13.1	0.76	0.4–1.4
Stomach (151)	39	47.0	0.83	0.6–1.1
Pancreas (157)	37	44.6	0.83	0.6–1.1
Kidney (180)	33	37.2	0.89	0.6–1.3
				
*Other specified sites*	422	521.3	0.81	0.7–0.9
Gallbladder	9	14.5	0.62	0.3–1.2
Ovary (175)	15	23.3	0.64	0.4–1.1
Multiple myeloma (203)	12	17.3	0.69	0.4–1.2
Other leukaemias (204, partly)	16	22.4	0.71	0.4–1.2
Prostate (177)	90	121.9	0.74	0.6–0.9
Non-Hodgkin's lymphoma (200, 202)	23	30.0	0.77	0.5–1.2
Colon (153)	120	142.2	0.84	0.7–1.0
Endometrium (172)	23	26.6	0.87	0.6–1.3
Rectum (154)	63	70.5	0.89	0.7–1.1
Brain (193)	31	23.5	1.32	0.9–1.9
Remaining specified sites	20	29.1	0.69	0.4–1.1
				
Secondary and unspecified sites	97	80.1	1.21	1.0–1.5

**Table 4:** Standardized incidence ratios (SIRs) for cancers at selected sites among 14 088 patients with Parkinson's disease by period of follow-up

	**Time since first hospitalisation (years)**			
	**<1**	**1–4**	**5–9**	**⩾10**
**Site of cancer (ICD-7)**	**SIR (n)**	**SIR (n)**	**SIR (n)**	**SIR (n)**
All sites combined (140–205)	0.99 (260)	0.89* (630)	0.80* (273)	0.78* (119)
Malignant melanoma (190)	2.35* (9)	2.18* (23)	1.66 (9)	1.11 (3)
Non-melanoma skin (191)	1.10 (44)	1.29* (143)	1.34* (75)	1.10 (30)
Female breast (170)	1.10 (20)	1.16 (62)	1.24 (36)	1.72* (24)
Lung (162.0–1)	0.42* (14)	0.39* (33)	0.23* (9)	0.59 (10)
Urinary bladder (181)	0.67 (12)	0.60* (28)	0.32* (7)	0.32* (3)
Prostate (177)	1.12 (27)	0.61* (37)	0.80 (21)	0.46 (5)
Colon (153)	1.13 (28)	0.92 (63)	0.65* (22)	0.46* (7)
Rectum (154)	1.00 (13)	0.99 (34)	0.68 (11)	0.72 (5)
Brain (193)	1.46 (6)	1.62 (18)	0.90 (5)	0.75 (2)
				
Secondary and unspecified sites^a^	1.85* (15)	1.42 (32)	0.87 (10)	0.72 (4)

* *P*<0.05.

a ICD-7 codes 198 and 199.
